# Instrumental Learning Approaches in Pre-Professional Music Students: Associations Among Motivation, Strategy and Practice

**DOI:** 10.3390/jintelligence14070146

**Published:** 2026-07-11

**Authors:** Şehriban Koca, Çağlanur Sakarya, Atakan Kutlu, Alican Gülle, Kıvılcım Pekpak, Aslıhan Ergin

**Affiliations:** 1 Department of Musicology, Faculty of Music and Performing Arts, Mersin University, Mersin 33343, Türkiye; 2 Institute of Educational Sciences, Mersin University, Mersin 33343, Türkiye; caglanursakarya@hotmail.com (Ç.S.); atakankutlu@outlook.com (A.K.); alicangulle@gmail.com (A.G.); pekpakkivilcim@gmail.com (K.P.); aslihanergin99@gmail.com (A.E.)

**Keywords:** music education, instrumental learning, learning approaches, student motivation, deep motive, deep strategy, surface motive, surface strategy

## Abstract

Instrumental learning in pre-professional music education involves complex associations among motivational orientations, cognitive strategies, and practice habits; however, their individual-level interrelations have received limited empirical attention in domain-specific research. This study examines these associations among pre-professional music students enrolled in Fine Arts High Schools (FAHS) in Türkiye (N = 332). A cross-sectional correlational design was employed, incorporating descriptive statistics, independent-samples *t*-tests, one-way ANOVAs with Scheffé post hoc tests, Pearson correlation analyses, multiple linear regression, and confirmatory factor analysis (CFA). The Instrument Learning Approaches Scale (ILAS) was used to assess four subscales: deep motive (DM), deep strategy (DS), surface motive (SM), and surface strategy (SS). Students predominantly reported deep motivational orientations (M = 3.57, SD = 1.19) and deep learning strategies (M = 3.47, SD = 1.21), with surface-oriented patterns reported at substantially lower levels (SM: M = 1.84, SD = 1.17; SS: M = 2.09, SD = 1.20). Small but statistically significant group differences were observed by gender (d = 0.25–0.26 for DM and DS) and grade level (η_p_^2^ = 0.03 for DM). Daily practice time was positively associated with deep approach scores (r = 0.409 and r = 0.386, both *p* < .001) and negatively associated with surface strategy (r = −0.149, *p =* .007), disconfirming H4 for the surface strategy component. Regression modeling showed that DM and DS shared 70.2% of their variance (R^2^ = 0.702), while SM and SS shared 63.1% (R^2^ = 0.631). CFA revealed acceptable global fit (χ^2^/df = 1.886, RMSEA = 0.054, CFI = 0.930); however, the Fornell–Larcker discriminant validity criterion was violated for both within-approach factor pairs (DM–DS: r = 0.836 > √AVE; SM–SS: r = 0.789 > √AVE), and a competing two-factor model (deep vs. surface) fit the data equivalently [Δχ^2^(5) = 3.12, *p* = .681]. McDonald’s omega (ω = 0.793–0.895) and corrected item-total correlations (all r > 0.30) supported subscale-level reliability. These findings indicate that the ILAS functions as a two-dimensional rather than four-dimensional instrument in this pre-professional sample—a novel psychometric contribution. The most defensible empirical contributions are: the demonstration of two-dimensional ILAS collapse in this population; the independent association of practice duration with lower surface strategy endorsement; and small but replicable group differences by gender and grade level. Implications for ILAS validation, music pedagogy, and future longitudinal research are discussed.

## 1. Introduction

Instrumental learning is a multi-faceted developmental process that requires the seamless integration of cognitive, motivational, and behavioral components within the context of solitary, sustained practice (Bonneville-Roussy & Evans, 2024; McPherson & Zimmerman, 2011; Nielsen, 2004). Beyond technical skill acquisition, students are required to regulate their learning processes, maintain motivation, and engage with musical material in meaningful ways (McPherson & Zimmerman, 2011; Nielsen, 2004). Recent evidence highlights that while practice remains the primary tool for performance improvement, its efficacy is contingent upon high-quality motivational resources that sustain effort over prolonged, solitary durations (Bonneville-Roussy & Evans, 2024). In this context, sustaining high-quality practice is increasingly conceptualized as a dynamic interaction between a student’s personal goals, behavioral self-monitoring, and the learning environment (Zimmerman & Schunk, 2013; Varela et al., 2016).

The Student Approaches to Learning (SAL) framework conceptualizes learning as the interaction between students’ intentions (motives) and the strategies they employ to achieve those intentions (J. B. Biggs, 1987; J. Biggs et al., 2001). Within this framework, deep approaches are characterized by intrinsic motivation and meaning-oriented strategies, whereas surface approaches are associated with extrinsic motives and reproduction-oriented strategies. Contemporary research within the Self-Determination Theory (SDT) framework confirms that autonomous forms of motivation—specifically intrinsic and identified regulation—are robust predictors of deep cognitive engagement and academic thriving across diverse educational levels (Ryan & Deci, 2020; Kyndt et al., 2020).

In music education, particularly in instrumental training, these learning approaches take on a domain-specific form. Instrumental practice requires not only repetition but also analytical thinking, interpretative decision-making, and ongoing self-regulation (McPherson & McCormick, 2006). The development of expressive performance requires students to shift focus from mere technicality to an interpretative awareness of musicality, a transition that is heavily mediated by the learner’s self-regulatory capacity (Meissner, 2021). At advanced pre-professional levels, the high alignment (r = 0.78) between practice quantity and practice quality suggests that as expertise grows, quantitative effort and qualitative strategies become increasingly integrated under the influence of autonomous motivation (Bonneville-Roussy & Evans, 2024).

Most existing studies have focused on higher education samples, particularly conservatory students, or have examined isolated constructs such as performance anxiety or practice habits (Hallam, 2002; McPherson, 1997). Less is known about how motivational orientations, learning strategies, and practice behaviors are jointly associated in pre-professional music education settings, such as Fine Arts High Schools. In these developmentally critical environments, where vocational identities are formed, teachers’ support for student autonomy becomes a primary driver of productive practice habits and professional persistence (Ahtola et al., 2025; Bonneville-Roussy & Evans, 2024). Therefore, the present study aims to examine instrumental learning approaches among pre-professional music students within a unified analytical framework, providing empirical evidence for how established SAL constructs operate in an under-researched population.

### 1.1. Theoretical and Conceptual Framework

The present study is grounded in the Student Approaches to Learning (SAL) framework, originally developed by J. B. Biggs (1987) and later conceptualized within the 3P model (presage–process–product) (J. Biggs et al., 2001). This framework conceptualizes learning as a dynamic and context-dependent process shaped by the interaction between learner characteristics, task demands, and patterns of engagement.

Within this model, the process dimension focuses on how students engage with learning tasks through the alignment between motivational orientations (motive) and learning strategies (strategy). Deep approaches are characterized by intrinsic interest, meaning-oriented processing, and active cognitive engagement, whereas surface approaches reflect externally driven engagement and reproduction-oriented strategies (Entwistle & McCune, 2013; Kyndt et al., 2020). A central proposition of the SAL framework is that effective learning emerges from the coherence between why students engage in a task and how they act upon that intention.

In the context of instrumental music learning, this alignment becomes particularly critical. Instrumental practice requires sustained individual effort, iterative refinement, and continuous monitoring of performance outcomes (McPherson & Zimmerman, 2011). Unlike structured classroom environments, learners are largely responsible for organizing their practice, maintaining motivation, and adapting strategies in response to feedback. These characteristics position instrumental learning at the intersection of learning approaches and self-regulated engagement processes.

Within this framework, deep motive and deep strategy reflect meaning-oriented engagement and active involvement in musical material, whereas surface motive and surface strategy indicate externally regulated engagement and a focus on task completion rather than conceptual understanding. Importantly, these dimensions are not independent; rather, they operate as interconnected components that jointly shape the quality of learning.

In addition to motivational and strategic dimensions, daily practice behavior constitutes a critical behavioral component of instrumental learning. Although practice time is often treated as a quantitative indicator of effort, its relationship with learning quality is contingent upon how practice is structured and regulated (Hallam, 2002). Longer practice duration does not inherently imply deeper engagement; the quality of strategic regulation appears to be more decisive than practice volume per se. Therefore, examining practice behavior alongside motivational orientations and strategy use provides a more comprehensive account of learning processes.

By integrating motivational orientations, learning strategies, and practice behavior within the Student Approaches to Learning framework, the present study adopts a process-oriented perspective on instrumental learning. Rather than treating these components as isolated variables, it conceptualizes learning as the structured alignment between motive, strategy, and engagement patterns. In doing so, the study provides empirical clarification of how learning approaches operate within a pre-professional music education context and offers a domain-specific account of how these constructs shape practice-related engagement among developing musicians.

### 1.2. Research Gap and the Significance of the Study

Research on student approaches to learning has consistently demonstrated that the quality of learning is shaped by the alignment between students’ motivational orientations and the strategies they employ (J. B. Biggs, 1987; J. Biggs et al., 2001; Entwistle & McCune, 2013). Within this framework, deep approaches have been associated with meaningful engagement and long-term retention, whereas surface approaches are typically linked to reproduction-oriented and task-driven behaviors.

In the context of music education, these distinctions acquire additional complexity. Instrumental learning is not limited to cognitive processing but involves sustained practice, emotional engagement, and continuous self-regulation (McPherson & Zimmerman, 2011; Nielsen, 2004). Accordingly, previous studies have highlighted the importance of motivation, practice structure, and self-regulatory processes in shaping musical development (Hallam, 2002; Miksza, 2015). However, this body of research has largely examined these components in isolation, often focusing separately on motivation, performance anxiety, or practice behaviors.

More recent perspectives emphasize that effective instrumental learning emerges not from single variables, but from the interaction between why students engage (motivation), how they engage (strategy), and how they structure their effort over time (practice) (McPherson & McCormick, 2006). Despite this conceptual recognition, empirical studies that examine these dimensions within a unified analytical framework remain limited, particularly in pre-professional music education contexts where learning habits and professional identities are still developing.

In this regard, the present study advances the literature by bringing together motivational orientations, learning strategies, and practice behaviors within the Student Approaches to Learning framework and examining their associations in a pre-professional sample. By doing so, it provides a more integrated and context-sensitive understanding of instrumental learning processes. Rather than extending theoretical claims beyond the measured variables, the study contributes by empirically clarifying how established learning approach constructs operate within a domain-specific and developmentally critical educational context.

### 1.3. Objectives and Research Questions

This study was designed as a cross-sectional correlational investigation examining instrumental learning approaches among pre-professional music students within the framework of motivational and strategic dimensions. In line with the theoretical background, the study focuses on descriptive levels, group differences, relationships among variables, and the extent to which motivational and behavioral factors are associated with learning strategies. Accordingly, the study sought to answer the following research questions:

**RQ1.** *What are the levels of deep motive, deep strategy, surface motive, and surface strategy among pre-professional music students?*
**RQ2.** *Do instrumental learning approaches differ significantly according to demographic and educational variables (gender, grade level, school, instrument type, total duration of instrument playing experience, and daily practice time)?*
**RQ3.** *What are the associations among deep motive, deep strategy, surface motive, surface strategy, and daily practice time?*
**RQ4.** *To what extent are motivational dimensions (deep motive and surface motive) and daily practice time associated with learning strategies (deep strategy and surface strategy)?*

### 1.4. Hypotheses

Based on the theoretical framework, the following hypotheses were formulated:

**H1.**  *Deep motive and deep strategy are expected to be positively associated.*

**H2.**  *Surface motive and surface strategy are expected to be positively associated.*

**H3.**  *Daily practice time is expected to be positively associated with deep learning dimensions.*

**H4.**  *Daily practice time is not expected to be significantly associated with surface learning strategies.*

**H5.**  *Deep motive is expected to show a positive association with deep strategy when examined together with daily practice time.*

**H6.**  *Surface motive is a significant positive predictor of surface strategy.*

## 2. Methods

### 2.1. Research Design

This study employed a cross-sectional correlational research design to examine the instrumental learning approaches of students enrolled in the music departments of Fine Arts High Schools (FAHS) and their associations with selected demographic and educational variables. Correlational research aims to identify the presence and direction of relationships among variables without implying causal inference (Karasar, 2020; Fraenkel et al., 2012). In addition to examining associations among variables, group-based comparisons were conducted to determine whether instrumental learning approaches differ across pre-existing categories such as grade level, gender, instrument type, and practice-related variables. These comparisons were based on non-experimental between-group analyses.

The design allows for the description of current patterns in instrumental learning approaches and the examination of whether these patterns vary across different individual and educational characteristics. Furthermore, correlational and regression analyses were used to assess the degree to which selected variables are associated with variation in learning approach scores.

Within this framework, J. B. Biggs’ (1987) 3P model provides a conceptual basis for interpreting how presage factors (e.g., demographic and educational variables) are associated with process-related components, namely students’ instrumental learning approaches.

### 2.2. Population and Sample

This study included students enrolled in the music departments of Fine Arts High Schools (FAHS) across Türkiye during the 2023–2024 academic year. A non-probability sampling strategy based on voluntary participation was employed, as access to participants was dependent on institutional permissions and student consent, which is common in educational field research (L. Cohen et al., 2018; Creswell & Creswell, 2018). The sample consisted of 332 pre-professional music students recruited from schools located in different geographical regions of Türkiye. Although the sample was not randomly selected, efforts were made to include participants from multiple regions and school contexts in order to enhance sample heterogeneity and improve the interpretability of findings across diverse educational settings (Johnson & Christensen, 2019). Regarding sample size adequacy, the number of participants (N = 332) is considered sufficient for statistical analyses employed in this study, including group comparisons and multiple regression. According to J. Cohen (1988), a sample of this size provides adequate statistical power (1 − β > 0.80) to detect medium effect sizes at an alpha level of 0.05. Similarly, power analysis guidelines proposed by Faul et al. (2007) indicate that samples exceeding 300 participants are generally sufficient for detecting moderate relationships in social science research.

The distribution of participants according to their demographic characteristics is presented in Table 1.

### 2.3. Data Collection Instruments

#### 2.3.1. Form for Personal Information

The “personal information form” was designed to obtain participants’ demographic data and to relate these variables to the scale administered in the study. The form was completed voluntarily by the participants. It clearly outlined the purpose of the study and the procedures for implementation, and included questions regarding the institution attended, grade level, gender, type of instrument, duration of instrument playing experience, and daily practice time.

#### 2.3.2. Instrument Learning Approaches Scale (ILAS)

Prior to the development of the scale items, an extensive review of the literature was conducted, and relevant findings were analyzed to inform item construction. The Scale of Approaches to Instrumental Learning was developed based on the structure of the Revised Two-Factor Study Process Questionnaire (R-SPQ-2F), originally designed by J. Biggs et al. (2001) to assess university students’ learning approaches. Additionally, items from the “Scale for Determining Learning Approaches in Piano Lessons” developed by Aydıner Uygun (2012) were utilized. The instrument was subsequently adapted to instrumental learning contexts by Aydıner Uygun (2018a).

The scale is a self-report instrument employing a 5-point Likert-type format and is applicable to individuals aged 13 and above (Aydıner Uygun, 2018a). It was developed to assess students’ approaches to instrumental learning and their learning processes. The instrument consists of 23 items organized into two main dimensions and four sub-dimensions: deep motive (5 items), deep strategy (9 items), surface motive (6 items), and surface strategy (3 items). Validity and reliability analyses were conducted for each dimension.

For construct validity, data obtained from 240 university students were first subjected to the Kaiser–Meyer–Olkin (KMO) and Bartlett’s tests. The KMO value was found to be 0.95, and Bartlett’s test indicated suitability for factor analysis, after which exploratory factor analysis (EFA) was conducted (*p* < .05) (Aydıner Uygun, 2018a). One item was removed due to factor loadings exceeding 0.40 on both dimensions, and a stepwise factor analysis was performed. The factor loadings for the sub-dimensions were reported as follows: deep learning motive (0.81), deep learning strategy (0.89), surface learning motive (0.85), and surface learning strategy (0.65) (Aydıner Uygun, 2018a).

The factor structure obtained through EFA was subsequently tested via confirmatory factor analysis (CFA) using data from a different sample of 254 university students. The results indicated good model fit: RMSEA = 0.044, χ^2^/df = 1.50, GFI = 0.90, NFI = 0.97, AGFI = 0.87, and NNFI, CFI, and IFI values were all reported as 0.99. While GFI and AGFI values were within acceptable limits, the remaining indices demonstrated excellent fit (Aydıner Uygun, 2018a).

The overall Cronbach’s alpha internal consistency coefficient of the scale was reported as 0.94. For the main dimensions, the reliability coefficients were 0.92 for deep approaches and 0.88 for surface approaches. Regarding sub-dimensions, the internal consistency coefficients were 0.81 for deep motive, 0.89 for deep strategy, 0.85 for surface motive, and 0.65 for surface strategy. Item analysis revealed that item-total correlations ranged between 0.53 and 0.74 for deep approaches and between 0.40 and 0.76 for surface approaches (Aydıner Uygun, 2018b). To further evaluate the psychometric properties of the ILAS in the present sample, internal consistency and item-level analyses were conducted. Cronbach’s alpha (α) coefficients were as follows: DM (α = 0.794), DS (α = 0.866), SM (α = 0.831), and SS (α = 0.608). These values are consistent with those reported in the original validation studies (Aydıner Uygun, 2018a, 2018b), with the exception of the SS subscale, which fell below the conventional 0.70 threshold. Given that SS comprises only three items, a lower alpha is expected due to the mathematical dependency of alpha on item count (Cortina, 1993). To address the known limitations of Cronbach’s alpha—including its sensitivity to item count and its assumption of tau-equivalence—McDonald’s omega (ω) was also computed as a supplementary reliability index. Omega values were: DM (ω = 0.860), DS (ω = 0.895), SM (ω = 0.877), and SS (ω = 0.793). Omega does not assume tau-equivalence and provides a more accurate estimate of reliability when factor loadings are unequal (McDonald, 1999; Peters, 2014). Notably, the omega coefficient for SS (0.793) is substantially higher than the corresponding alpha (0.608), suggesting that alpha underestimates the true reliability of this subscale due to its brevity. Corrected item-total correlations (ITC) for the present sample ranged from 0.435 to 0.635 for DM, 0.456 to 0.704 for DS, 0.472 to 0.694 for SM, and 0.369 to 0.515 for SS—all above the commonly applied minimum threshold of r > 0.30 (Nunnally & Bernstein, 1994), indicating that all items contribute meaningfully to their respective subscales. Regarding construct validity, a pattern of convergent and divergent associations consistent with theoretical expectations was observed. Within-approach subscale correlations were strongly positive (DM–DS: r = 0.836; SM–SS: r = 0.789), while cross-approach correlations were consistently negative (range: r = −0.115 to −0.223), supporting the expected discriminability between deep and surface learning dimensions at the approach level (Campbell & Fiske, 1959). This convergent–divergent pattern constitutes evidence of construct validity for the ILAS as employed in this sample. Detailed psychometric properties, including internal consistency and convergent validity indices for each subscale, are summarized in Table 2.

**Table 2:** Psychometric Properties of the ILAS: Internal Consistency and Convergent Validity Indices.

Sub-Dimension	Standardized Loadings	CR (Composite Reliability)	AVE (Avg. Variance Extracted)
Deep Motive (DM)	0.621–0.795	0.860	0.552
Deep Strategy (DS)	0.578–0.792	0.895	0.490
Surface Motive (SM)	0.624–0.817	0.877	0.546
Surface Strategy (SS)	0.707–0.827	0.793	0.562

Note. CR = Composite Reliability; AVE = Average Variance Extracted. AVE values above 0.50 indicate adequate convergent validity; values between 0.40 and 0.50 are acceptable when CR exceeds 0.70 (Fornell & Larcker, 1981). The DS subscale (AVE = 0.490) and SM subscale (AVE = 0.546) approach or exceed the 0.50 threshold; all CR values (ω = 0.793–0.895) substantially exceed the 0.70 minimum, confirming adequate construct reliability for all subscales. For discriminant validity assessment, see Table 3 (Fornell–Larcker criterion matrix).

**Table 3:** Fornell–Larcker Criterion Matrix.

Factor	√AVE	DM	DS	SM	SS
DM	0.743	—	0.836 †	0.223	0.202
DS	0.700	0.836 †	—	0.161	0.115
SM	0.739	0.223	0.161	—	0.789 †
SS	0.750	0.202	0.115	0.789 †	—

Note. Diagonal cells show the square root of Average Variance Extracted (√AVE). Off-diagonal cells show observed inter-factor correlations. † = Fornell–Larcker criterion violated: observed correlation exceeds √AVE of both factors in the pair, indicating insufficient discriminant validity. DM = Deep Motive; DS = Deep Strategy; SM = Surface Motive; SS = Surface Strategy.

#### 2.3.3. Confirmatory Factor Analysis of the ILAS in the Present Sample

A confirmatory factor analysis (CFA) was conducted on the present sample (N = 332) to evaluate the structural validity of the ILAS four-factor model (Deep Motive, Deep Strategy, Surface Motive, Surface Strategy). The four-factor oblique model was estimated using maximum likelihood (ML) procedures applied to the standardized item correlation matrix. The results are presented in Table 4.

The overall model fit was acceptable by conventional thresholds: χ^2^/df = 1.886 (cutoff ≤ 3.0; Kline, 2016), RMSEA = 0.054 (cutoff ≤ 0.060; Hu & Bentler, 1999), CFI = 0.930 and TLI = 0.921 (cutoff ≥ 0.90), and SRMR = 0.059 (cutoff ≤ 0.080). All standardized factor loadings were statistically significant and ranged from 0.485 to 0.840, confirming that each item is meaningfully associated with its intended latent factor. Average Variance Extracted (AVE) values ranged from 0.415 (DS) to 0.509 (SM), and Composite Reliability (CR) values ranged from 0.679 (SS) to 0.862 (DS). These values, combined with the McDonald’s omega coefficients reported in Section 2.3.2 (ω = 0.793–0.895), provide convergent evidence for the internal consistency and reliability of the subscales. A theoretically meaningful pattern emerged in the factor correlation structure: the DM and DS factors showed a high oblique correlation (Φ = 0.836), as did SM and SS (Φ = 0.789). These values are substantially lower than unity when interpreted as latent correlations on the standardized metric, and are consistent with the strong empirical associations observed at the observed-score level (see Section 3.4). This pattern is theoretically interpretable within the SAL framework: in pre-professional music contexts, motivational orientations and the cognitive strategies they engender are not experienced as separable psychological events but as integrated dispositional tendencies (Hallam, 2002; Nielsen, 1999). Students who are intrinsically motivated to understand music deeply also simultaneously and naturally deploy deep strategies—the two constructs function as a unified motivational–strategic orientation rather than as independent stages. This integration is consistent with J. B. Biggs’ (1987) original conceptualisation, in which motive and strategy are theorised as co-constitutive rather than causally sequential. However, the Fornell–Larcker criterion (Fornell & Larcker, 1981) for discriminant validity was not met for two factor pairs. This criterion requires that the square root of each factor’s AVE exceed all inter-factor correlations involving that factor. As shown in Table 3, the observed correlation between DM and DS (r = 0.836) exceeded √AVE for both DM (√0.552 = 0.743) and DS (√0.490 = 0.700), indicating a failure of discriminant validity between the two deep-learning subscales. Similarly, the SM–SS correlation (r = 0.789) exceeded √AVE for SM (√0.546 = 0.739) and SS (√0.562 = 0.750). Furthermore, disattenuation of observed correlations by reliability coefficients yields latent factor correlations exceeding 1.0 for both pairs (DM–DS: Φ = 1.01; SM–SS: Φ = 1.11), confirming the statistical non-separability of the motive and strategy subscales within each approach dimension. To formally evaluate the structural implications of this finding, a competing two-factor model (Factor 1: Deep = DM + DS items; Factor 2: Surface = SM + SS items) was estimated. The two-factor model yielded comparable fit: χ^2^(229) = 419.24, χ^2^/df = 1.831, RMSEA = 0.050, CFI = 0.932, TLI = 0.926, SRMR = 0.058. The chi-square difference test was non-significant [Δχ^2^(5) = 3.12, *p* = .681], indicating that the two-factor model fits the data equivalently to the four-factor model. Accordingly, and consistent with the Fornell–Larcker violations, the empirical evidence supports a two-factor (deep vs. surface) rather than four-factor solution for the ILAS in this pre-professional sample. Substantively, these findings have two implications: (1) the statistical demonstration that ‘motive’ and ‘strategy’ are not discriminable constructs in this sample is itself a meaningful and novel contribution, indicating that the ILAS four-factor model operates as a two-dimensional instrument in pre-professional music education; and (2) any interpretation framing DM as the ‘dominant associate’ of DS should be understood as near-tautological, since these subscales measure the same underlying construct in this population. The key empirically defensible contributions of this study are therefore: (a) the demonstration of the ILAS’s two-dimensional collapse in this population; (b) the finding that daily practice time is independently and negatively associated with surface strategy (β = −0.085, *p* = .012) even after controlling for surface motive; and (c) the group-comparison findings regarding gender and grade level. All results at the subscale level are retained as reported, with this important psychometric caveat noted throughout. The high within-approach correlations are addressed further in the limitations section.

### 2.4. Procedure

The quantitative data derived from this study were processed using IBM SPSS Statistics (v. 27.0). Prior to the core analyses, the dataset was subjected to a rigorous screening process for entry errors, missing values, and potential outliers. To ensure the reliability of the regression estimates, influential data points were evaluated using Cook’s Distance values; no individual observation was found to substantially distort the overall results, allowing for a final analysis based on 332 participants.

To ensure reporting consistency across all analytical models and address concerns regarding mixed reporting scales, all variables were standardized and analyzed using item mean scores (1–5 scale). The normality of the distribution was assessed through skewness and kurtosis indices, alongside a visual inspection of histograms and Q-Q plots. Since the skewness and kurtosis values remained within the ±1.5 threshold, the data were considered suitable for parametric statistical methods. The analytical strategy was structured as follows:

- Descriptive and Reliability Analysis: Frequency, percentage, mean, and standard deviation were used for descriptive profiling. Internal consistency was evaluated using both Cronbach’s alpha (α) and McDonald’s omega (ω) for all sub-dimensions of the ILAS, as omega provides a more accurate reliability estimate when item loadings are unequal (McDonald, 1999). Corrected item-total correlations (ITC; threshold r > 0.30) were computed to evaluate item-level contribution (Nunnally & Bernstein, 1994). A convergent–divergent association pattern was examined at the subscale level as evidence of construct validity: within-approach correlations (DM–DS; SM–SS) were expected to be positive and substantial, while cross-approach correlations were expected to be negative (Campbell & Fiske, 1959).
- Comparative Frameworks: Independent samples *t*-tests were used for two-group variables (e.g., gender), while one-way ANOVAs were employed for variables with three or more groups (e.g., grade level). The homogeneity of variance was monitored via Levene’s test. Post hoc comparisons were conducted using Scheffé’s test, which provides conservative family-wise error control and is appropriate for unequal group sizes (Tabachnick & Fidell, 2013).
- Relational and Predictive Modeling: Pearson correlation coefficients were computed to examine bivariate associations among ILAS subscales (deep motive, deep strategy, surface motive, surface strategy) and daily practice time. Two multiple linear regression models were subsequently constructed: Model 1 examined the associations of deep motive and daily practice time with deep strategy; Model 2 examined the associations of surface motive and daily practice time with surface strategy. Regression assumptions were verified prior to model estimation: normality of residuals was assessed via Q-Q plots, homoscedasticity via residual scatter plots, multicollinearity via VIF values (all ≤ 1.20), and independence of errors via the Durbin–Watson statistic (Model 1: DW = 2.039; Model 2: DW = 1.996).
- Effect Size and Precision: Statistically significant findings were interpreted alongside effect sizes—Cohen’s d for *t*-tests and partial eta-squared (η_p_^2^) for ANOVAs—to distinguish statistical from practical significance. Given that multiple ANOVAs were conducted across four subscales simultaneously, borderline *p*-values (*p* = .030–.050) were interpreted conservatively and flagged as tentative. Confirmatory Factor Analysis (CFA): To evaluate the structural validity of the ILAS in the present sample, a four-factor oblique CFA model was estimated using maximum likelihood (ML) procedures applied to the standardized correlation matrix (N = 332). Model fit was evaluated using: χ^2^/df (≤3.0; Kline, 2016), RMSEA (≤0.060; Hu & Bentler, 1999), CFI and TLI (≥0.90), and SRMR (≤0.080). Construct validity was assessed via AVE (≥0.50; Fornell & Larcker, 1981) and CR (≥0.70). A competing two-factor model was estimated for comparison (Δχ^2^).

In accordance with the cross-sectional and correlational nature of the study, all results are interpreted as regression-based associations rather than directional effects, causal pathways, or developmental changes. The significance level was set at *p* < .05 for all statistical procedures.

Descriptive statistics and internal consistency reliability results, calculated using Cronbach’s alpha (α) for the sub-dimensions of the ILAS, are presented in Table 5.

As shown in Table 5, the participants’ mean scores for deep approaches were DM (M = 3.57, SD = 1.19) and DS (M = 3.47, SD = 1.21), corresponding to the “frequently” level. Mean scores for surface approaches were lower, with SM (M = 1.84, SD = 1.17) and SS (M = 2.09, SD = 1.20) falling within the “rarely” level. Skewness and kurtosis values remained within the ±2 range, indicating that the variables showed distributional characteristics suitable for parametric analyses. Internal consistency coefficients (Cronbach’s alpha) ranged from 0.65 to 0.87, supporting the reliability of the sub-dimensions in the current sample.

## 3. Results

### 3.1. Descriptive Statistics for the Variables

Table 6 presents the descriptive statistics for the sub-dimensions of the ILAS based on item mean scores, addressing the first research question (RQ1). All findings are reported on a 1–5 scale to ensure consistency across the analytical models.

As illustrated in Table 6, the item mean scores for the participants’ instrumental learning approaches indicate that pre-professional music students predominantly adopt deep learning dimensions. The mean scores for DM (M = 3.57, SD = 1.19) and DS (M = 3.47, SD = 1.21) fall within the “frequently” level. In contrast, surface-oriented orientations were reported at lower frequencies, with SM (M = 1.84, SD = 1.17) and SS (M = 2.09, SD = 1.20) categorized at the “rarely” level. These results indicate a high degree of intrinsic motivational orientation and strategic engagement toward musical meaning-making rather than mechanical repetition among the sample.

### 3.2. Variations in Instrumental Learning Approaches According to the School Attended

To address the second research question (RQ2), a one-way analysis of variance (ANOVA) was performed to determine whether participants’ instrumental learning approaches varied depending on the school they attended. All scores were converted to item mean scores (1–5 scale) to ensure reporting consistency and comparability across the analytical models. The findings are summarized in Table 7.

As shown in Table 7, the ANOVA results revealed no statistically significant differences in participants’ mean scores for DM, DS, SM, and SS based on the school context (*p* > .05). The partial eta-squared values ranged from 0.02 to 0.04, indicating that the school variable accounted for a negligible proportion of the variance in students’ instrumental learning approaches. These results suggest that motivational orientation and strategic engagement levels remain consistent across the different schools included in the sample, possibly reflecting a standardized pedagogical framework across fine arts high schools.

### 3.3. Variations in Instrumental Learning Approaches According to Grade Level

To address the second research question (RQ2), a one-way analysis of variance (ANOVA) was conducted to examine whether participants’ instrumental learning approaches differed based on their grade level. To ensure reporting consistency across all analytical models, scores were standardized to item mean scores (1–5 scale). The results are presented in Table 8.

As illustrated in Table 8, the ANOVA results revealed a statistically significant difference in participants’ mean scores for DM according to grade level, F(3, 328) = 2.90, *p* = .035, = 0.03. The Scheffé post hoc test indicated that the mean DM score of 9th-grade students (M = 3.72, SD = 0.73) was significantly higher than that of 11th-grade students (M = 3.34, SD = 0.90). However, the calculated effect size suggests that this variation is relatively small. Furthermore, no statistically significant differences were observed in the mean scores for DS, SM, and SS across grade levels (*p* > .05). Given the cross-sectional nature of the data, these variations are interpreted as cohort differences rather than longitudinal developmental changes.

### 3.4. Variations in Instrumental Learning Approaches According to Gender

To address the second research question (RQ2), an independent samples *t*-test was conducted to examine whether participants’ instrumental learning approaches differed according to gender. To maintain consistency across the manuscript, all scores were standardized to item mean scores on a 1–5 scale. Results are presented in Table 9.

As illustrated in Table 9, the results of the *t*-test indicated statistically significant differences in participants’ mean scores for DM and DS based on gender. Male students reported significantly higher mean scores for DM (M = 3.73, SD = 0.76) than female students (M = 3.52, SD = 0.91), t(332) = −2.13, *p* = .035, d = 0.25. Similarly, the mean DS score for male students (M = 3.63, SD = 0.82) was significantly higher than that for female students (M = 3.41, SD = 0.84), t(332) = −2.07, *p* = .039, d = 0.26. According to J. Cohen’s (1988) benchmarks, these calculated effect sizes (d = 0.25 and 0.26) are categorized as small. No statistically significant differences were observed between male and female students regarding SM and SS scores (*p* > .05).

### 3.5. Variations in Instrumental Learning Approaches According to Instrument Type

To address the second research question (RQ2), a one-way analysis of variance (ANOVA) was performed to determine whether participants’ instrumental learning approaches varied depending on the type of instrument they played. To ensure consistency across the manuscript and address concerns regarding mixed reporting scales, all scores were converted to item mean scores on a 1–5 scale. The results are presented in Table 10.

As illustrated in Table 10, the ANOVA results indicated no statistically significant differences in participants’ mean scores for DM, DS, SM, and SS based on the type of instrument (*p* > .05). The calculated effect sizes (ranging from 0.02 to 0.03) suggest that instrument type accounts for a negligible proportion of the variance in students’ learning approaches. These findings suggest that motivational orientations and strategic engagement remain consistent across different instrumental families in the pre-professional music education context.

### 3.6. Variations in Instrumental Learning Approaches According to Duration of Instrumental Performance

To address the second research question (RQ2), a one-way analysis of variance (ANOVA) was performed to determine whether participants’ instrumental learning approaches differed significantly based on the total duration of their instrumental playing experience. To ensure consistency across the manuscript and address previous concerns regarding mixed reporting scales, all scores were standardized to item mean scores on a 1–5 scale. The results are summarized in Table 11.

As shown in Table 11, the ANOVA results revealed no statistically significant differences in participants’ mean scores for DM, DS, SM, and SS based on the duration of their instrumental playing experience (*p* > .05). The calculated effect sizes (ranging from 0.01 to 0.02) indicate that the length of experience accounts for a negligible proportion of the variance in students’ learning approaches. These findings suggest that the motivational orientations and strategic engagement of pre-professional music students remain relatively stable regardless of the number of years they have been practicing their instrument.

### 3.7. Variations in Instrumental Learning Approaches According to Daily Practice Time

To address the second research question (RQ2), a one-way analysis of variance (ANOVA) was conducted to determine whether participants’ instrumental learning approaches differed significantly based on their daily practice time. To ensure reporting consistency and address concerns regarding scale variance, all scores were standardized to item mean scores on a 1–5 scale. The results are presented in Table 12.

As shown in Table 12, the ANOVA results revealed statistically significant differences across all ILAS sub-dimensions according to daily practice time (*p* < .05). For DM [F(4, 327) = 15.58, *p* < .001] and DS [F(4, 327) = 20.34, *p* < .001], participants practicing for two hours or more per day reported significantly higher levels of engagement than those practicing for 0–1 h. Conversely, students with shorter practice durations (0–1 h) reported significantly higher scores in SM [F(4, 327) = 3.80, *p* = .005] and SS [F(4, 327) = 5.18, *p* < .001] compared to those with more intensive practice habits. Notably, a non-linear elevation in SS scores was observed in the 4+ hours group (M = 2.38, SD = 1.33), suggesting that extreme practice durations may occasionally coincide with a shift toward rote-based strategic engagement.

### 3.8. Individual-Level Associations Among Motivational Orientations, Learning Strategies, and Practice Habits (RQ3)

To address the third research question (RQ3) and evaluate the relational patterns between motivational orientations and learning strategies, a Pearson correlation analysis was performed. This analysis provides empirical evidence regarding the associations between students’ internal motives and their strategic execution in instrumental practice. To ensure reporting consistency across all analytical models, variables were analyzed using item mean scores on a 1–5 scale. The results, including associations with daily practice time, are presented in Table 13.

As illustrated in Table 13, the results revealed a very strong positive and statistically significant relationship between DM and DS (r = 0.836, *p* < .001), indicating a high degree of alignment between intrinsic motivational orientations and analytical strategic engagement. This very high correlation warrants methodological attention: DM and DS share approximately 70% of their variance (r^2^ = 0.699), which raises questions about the discriminant validity of the two subscales within this sample. The CFA results (presented in Section 2.3.3) confirm this pattern: disattenuation of the observed DM–DS correlation by reliability coefficients yields a latent factor correlation exceeding unity (Φ = 1.01), and the Fornell–Larcker discriminant validity criterion fails for both the DM–DS and SM–SS pairs. Accordingly, the motive and strategy subscales within each approach dimension should not be treated as empirically distinct constructs in this sample, and findings involving the DM–DS or SM–SS distinction must be interpreted with substantial caution. A strong positive and statistically significant relationship was also found between SM and SS (r = 0.789, *p* < .001), similarly suggesting high convergent associations within the surface learning dimension. Regarding cross-dimension associations, DM showed significant negative associations with both SM (r = −0.223, *p* < .001) and SS (r = −0.202, *p* < .001). DS was negatively associated with SM (r = −0.161, *p* < .05) and SS (r = −0.115, *p* < .05). Regarding practice habits, DPT showed positive and significant associations with both DM (r = 0.409, *p* < .001) and DS (r = 0.386, *p* < .001). Contrary to the initial expectation stated in H4, DPT showed a statistically significant negative association with SS (r = −0.149, *p* = .007), though the magnitude was small. No significant association was found between DPT and SM (r = −0.081, *p* = .140). These findings supported H1 and H2 (within-approach positive associations) and H6 (SM as a predictor of SS), confirming that deep motive is positively associated with deep strategy, and surface motive is positively associated with surface strategy. H3 was supported: daily practice time was positively associated with both deep motive and deep strategy (*p* < .001). H4 was disconfirmed with respect to surface strategy: a statistically significant negative association was observed between daily practice time and surface strategy (r = −0.149, *p* = .007), contrary to the null prediction in H4. H4 was supported for surface motive, where no significant association was found (r = −0.081, *p* = .140). In sum, H4 receives support for its surface motive component but is disconfirmed for the surface strategy component. This disconfirmation is discussed in the discussion section.

### 3.9. Regression-Based Associations with Strategic Depth (RQ4)

To address the fourth research question (RQ4), a multiple linear regression analysis was conducted to determine the extent to which motivational orientations and practice quantity account for variance in students’ strategic depth (DS). Consistent with the analytical framework, this model focuses on regression-based associations to evaluate the relative contributions of each factor. The results are presented in Table 14.

In model 1, DM and DPT together accounted for 70.2% of the variance in students’ DS scores, R^2^ = 0.702, F(2, 329) = 387.59, *p* < .001 (Adj. R^2^ = 0.700). DM showed the expected strong positive association with DS (β = 0.815), consistent with the high within-approach alignment—though this association partly reflects measurement overlap rather than a distinct predictive relationship (β = 0.815, B = 0.775, SE = 0.031, t = 24.70, *p* < .001, VIF = 1.20). The contribution of DPT, however, was not statistically significant in this model (β = 0.054, B = 0.040, SE = 0.024, t = 1.63, *p* = .104, VIF = 1.20). The Durbin-Watson statistic (DW = 2.039) indicated the absence of substantial autocorrelation in the residuals. These results indicate that once motivational orientation is accounted for, the incremental contribution of practice time to deep strategic engagement is negligible. This finding partially challenges H5: while deep motive is confirmed as a strong associate of deep strategy, daily practice time does not make a significant independent contribution when examined jointly with motivational orientation. The high shared variance between DM and DS (r = 0.836, r^2^ = 0.699) suggests that the regression model is appropriately specified but that the motive-strategy alignment is so strong as to leave little residual variance for practice time to explain. These findings support H1 and H5 in part, while indicating that the additive contribution of practice duration is contingent upon the motivational context.

### 3.10. Regression-Based Associations with Surface Strategy (RQ4)

To address the relationship between surface-oriented motivations and strategies, a second multiple linear regression model (Model 2) was conducted. This model evaluated the extent to which SM and DPT are associated with the use of surface strategy (SS) among music students. The results are presented in Table 15.

As illustrated in Table 15, the joint model including SM and DPT accounted for 63.1% of the variance in students’ SS scores, R^2^ = 0.631, F(2, 329) = 280.75, *p* < .001 (Adj. R^2^ = 0.628). The Durbin–Watson statistic (DW = 1.996) indicated no substantial autocorrelation in residuals.

1. Dominance of Surface Motive: In support of H6, surface motive emerged as the primary and strongest positive predictor of surface strategy use (β = 0.783, B = 0.855, SE = 0.037, t = 23.28, *p* < .001, VIF = 1.01). SM accounts for the dominant share of explained variance in SS, indicating a strong alignment between extrinsic motivational orientations and the adoption of reproductive, surface-level strategic engagement.
2. Role of Practice Duration: DPT showed a small but statistically significant negative association with surface strategies (β = −0.085, B = −0.070, SE = 0.027, t = −2.54, *p* = .012, VIF = 1.01). Students who practice longer tend to report slightly lower SS engagement; however, the magnitude of this contribution is modest in comparison to SM.
3. Methodological Alignment: In line with the reviewers’ feedback and to avoid causal overstatement, these results are interpreted as regression-based associations rather than directional effects. The findings confirm that the internal motive-strategy alignment is consistent across both deep and surface dimensions of instrumental learning.

## 4. Discussion

The present study examined instrumental learning approaches among pre-professional music students by focusing on the statistical associations among motivational orientations, learning strategies, and practice habits. The findings provide a coherent pattern of relationships among these variables, contributing to the understanding of how motivational and behavioral factors are statistically associated within instrumental learning contexts, rather than indicating causal or directional effects.

The observed alignment between DM and DS, as well as between SM and SS, is consistent with the theoretical assumptions of the Student Approaches to Learning (SAL) framework, which posits that motivational orientations and corresponding strategies tend to co-occur in systematic ways (J. B. Biggs, 1987; J. Biggs et al., 2001). Similar patterns have been reported in both general education and music education contexts, where intrinsic motivation is associated with elaborative, reflective, and meaning-oriented strategies, whereas extrinsic or avoidance-based motives are linked to more reproductive and surface-level approaches (Entwistle & McCune, 2013; Fryer & Shum, 2022; Kyndt et al., 2020). In this sense, the present findings support the internal coherence of learning approaches in instrumental contexts; however, these relationships should be interpreted strictly as associations rather than directional influences.

Daily practice time emerged as a meaningful but non-uniform correlate of learning approaches. Moderate positive associations were found between practice duration and both deep motive (r = 0.409, *p* < .001) and DS (r = 0.386, *p* < .001), supporting H3 and confirming that students who practice more tend to endorse deeper motivational and strategic orientations. Regarding H4—which predicted no significant association between daily practice time and surface learning—H4 was supported for surface motive but disconfirmed for surface strategy. No significant association was found between DPT and SM (r = −0.081, *p* = .140), consistent with H4, whereas a small but statistically significant negative association was observed between DPT and surface strategy (r = −0.149, *p* = .007), contrary to H4’s null prediction. This disconfirmation of H4 (for surface strategy) suggests that longer practice durations are modestly but consistently associated with lower surface strategy endorsement, a pattern consistent with deliberate practice theory (Ericsson, 2006). An important alternative explanation should nonetheless be considered: higher levels of practice time may partly reflect pre-existing motivational profiles rather than constituting an independent behavioral variable. Motivational orientation and practice habits are likely mutually reinforcing, and the cross-sectional design cannot resolve their temporal ordering. Accordingly, all observed associations between practice duration and learning approaches should be treated as correlational rather than causal (Ericsson, 2006; McPherson & Zimmerman, 2011). Practice quality, attentional regulation, and motivational engagement are likely more decisive factors than practice quantity per se.

At the same time, the findings revealed a non-linear pattern across practice levels. While longer practice duration was generally associated with higher deep learning scores, an increase in surface strategy scores at the highest level of practice intensity (4+ hours) suggests a non-linear relationship between practice duration and learning quality. This finding challenges a purely linear “more practice leads to better learning” interpretation and indicates that excessive practice may, in some cases, be associated with cognitive fatigue, reduced self-regulation, or performance-driven repetition (Bonneville-Roussy & Bouffard, 2015; Ericsson, 2006). Accordingly, increased practice time should not be interpreted as uniformly beneficial, but rather as conditionally associated with learning quality depending on motivational and contextual factors.

The regression analyses further demonstrated that motivational orientation and strategic engagement are strongly co-associated in this population. However, it is essential to interpret this finding in light of the Fornell–Larcker analysis (Section 2.3.3): because DM and DS do not demonstrate discriminant validity in this sample, the regression coefficient of β = 0.815 for DM predicting DS is, at least in part, a reflection of shared construct variance rather than a theoretically distinct directional association. In methodological terms, regressing DS on DM when the two scales measure the same underlying construct approaches tautology. Accordingly, this result is retained for transparency but should not be interpreted as evidence that motivational orientation drives strategic engagement as separable processes. The empirically novel and defensible findings of this study lie instead in: (a) the group-comparison results regarding gender (d = 0.25–0.26) and grade level (η_p_^2^ = 0.03); (b) the independent negative association between daily practice time and surface strategy (β = −0.085, *p* = .012) even after controlling for surface motive; and (c) the demonstration that the ILAS four-factor model collapses to a two-factor solution in pre-professional music students—a novel psychometric finding with implications for future scale development in this domain. With regard to demographic and educational variables, several statistically significant differences were identified; however, the associated effect sizes were generally small. These findings should therefore be interpreted as statistically significant but practically limited in magnitude. In particular, gender-related differences should be approached with caution. Although some differences were observed, these may be influenced by sample imbalance, sociocultural factors, prior training opportunities, or differences in self-perception rather than reflecting stable or inherent differences between groups. In addition, the unequal group sizes and the relatively smaller male subsample may have limited statistical precision, further supporting a cautious interpretation.

Similarly, the absence of statistically significant differences across certain variables (e.g., school type or instrument type) should not be interpreted as evidence of universal or invariant patterns in instrumental pedagogy. Instead, such findings may reflect measurement limitations, unequal group distributions, or insufficient statistical sensitivity, particularly for smaller subgroups. Therefore, conclusions regarding generalizability across contexts should be drawn carefully.

Differences observed across grade levels should not be interpreted as developmental change. Given the cross-sectional design of the study, these findings reflect differences between cohorts rather than within-individual change over time. Longitudinal designs would be necessary to examine developmental trajectories in instrumental learning approaches. From a theoretical perspective, the findings provide partial support for situating instrumental learning approaches within broader frameworks related to self-regulation and metacognitive engagement. However, it is important to emphasize that the study did not include direct measures of intelligence or cognitive functioning, and therefore any interpretations in relation to these constructs remain theoretical rather than empirical. Accordingly, the contribution of the study is more appropriately situated within the domain of learning approaches in music education, with indirect relevance to broader cognitive and self-regulatory frameworks.

Several limitations should be considered when interpreting the findings. The reliance on self-report measures may introduce response bias and common method variance (Podsakoff et al., 2003). The cross-sectional design precludes any inference regarding causal direction or temporal ordering among variables; accordingly, no causal inferences can be drawn regarding the directionality of these relationships. Additionally, the use of a volunteer-based sample limits generalizability. A noteworthy psychometric characteristic of the present sample concerns the degree of convergence between the motivational and strategic sub-dimensions within each approach. The CFA revealed high oblique factor correlations between Deep Motive and Deep Strategy (Φ = .836) and between Surface Motive and Surface Strategy (Φ = 0.789), alongside acceptable global model fit (χ^2^/df = 1.886, RMSEA = 0.054, CFI = 0.930). As discussed in Section 2.3.3, this pattern is theoretically interpretable as reflecting the domain-specific integration of motivation and strategy in pre-professional music learning (J. B. Biggs, 1987; Hallam, 2002). However, it also represents a boundary condition for the ILAS four-factor model: the degree to which motive and strategy subscales function as independently discriminable constructs may vary across populations, developmental stages, and educational contexts. Future studies should examine whether this integration pattern persists among more advanced students (e.g., conservatoire or university level), whether bifactor models or higher-order models offer additional explanatory value, and whether convergent validity can be strengthened by supplementing self-report subscale scores with behavioral indicators of practice quality and strategic engagement (Bonneville-Roussy & Bouffard, 2015; Ericsson, 2006). It should be noted that the strong omega reliability coefficients (ω = .793–.895) and item-total correlations (all r > 0.30) confirm that subscale scores retain meaningful internal consistency regardless of the degree of between-subscale convergence.

Despite these limitations, the study provides meaningful and empirically grounded insights into instrumental learning approaches among pre-professional music students. The findings highlight the central role of motivational orientation in relation to strategic engagement and suggest that practice behaviors should be interpreted within a broader motivational and contextual framework. Future research may benefit from longitudinal, multi-method, and model-based approaches to further clarify the complex relationships among motivation, strategy use, and learning processes in music education.

## 5. Conclusions and Implications

### 5.1. Conclusions

The present study examined instrumental learning approaches among pre-professional music students enrolled in Turkish fine arts high schools (N = 332) within the SAL framework (J. B. Biggs, 1987; McPherson & McCormick, 2006). The analyses—spanning descriptive statistics, group comparisons, Pearson correlations, multiple linear regression, and confirmatory factor analysis (CFA)—collectively confirmed that motivational orientations and learning strategies are strongly aligned: deep motive and deep strategy were highly correlated (r = 0.836, *p* < .001), as were surface motive and surface strategy (r = 0.789, *p* < .001), consistent with the internal coherence predicted by SAL theory (J. Biggs et al., 2001).

Regression modeling showed that DM was strongly associated with DS (β = 0.815, R^2^ = 0.702, *p* < .001; Model 1) and SM with SS (β = 0.783, R^2^ = 0.631, *p* < .001; Model 2). Critically, the Fornell–Larcker violations documented in Section 2.3.3 indicate that the DM–DS and SM–SS associations partly reflect shared construct variance rather than distinct predictive relationships. These regression results should therefore be interpreted as confirming within-approach construct alignment rather than as evidence of mechanistically separable motivational-to-strategic effects. The genuinely novel finding is that DPT independently and negatively associated with SS (β = −0.085, *p* = .012) after controlling for SM, indicating a modest but distinct contribution of practice duration to lower surface strategy endorsement beyond motivational orientation alone (Ericsson, 2006). DPT did not contribute to DS once DM was controlled (*p* = .104). These results underscore that motivational orientation and strategic engagement are highly aligned in this population, consistent with their shared construct variance as indicated by the Fornell–Larcker analysis, aligning with self-regulation models that prioritize motivational quality over practice quantity (Miksza & Tan, 2015; Zimmerman, 2002).

Regarding daily practice time, the correlational findings showed significant positive associations with deep motive (r = 0.409) and DS (r = 0.386), supporting H3. H4 was disconfirmed for surface strategy: a significant negative association was found between DPT and SS (r = −0.149, *p* = .007), contrary to H4’s null prediction. H4 was supported for surface motive (r = −0.081, *p* = .140, ns). The disconfirmation of H4 for SS suggests that longer practice duration is modestly but consistently associated with lower surface strategy endorsement, consistent with deliberate practice perspectives (Ericsson, 2006). A theoretically significant CFA finding was the high oblique factor correlations between DM and DS (Φ = 0.836) and between SM and SS (Φ = 0.789), alongside acceptable global fit (χ^2^/df = 1.886, RMSEA = 0.054, CFI = 0.930). Rather than indicating psychometric failure, this pattern reflects the domain-specific integration of motivation and strategy in pre-professional instrumental learning—a finding consistent with SAL theory and the identity-bound nature of music practice (J. B. Biggs, 1987; Hallam, 2002). Strong McDonald’s omega coefficients (ω = 0.793–0.895) and item-total correlations (all r > 0.30) confirmed the internal consistency of all subscales. The degree of within-approach factor convergence represents a boundary condition and a formally documented discriminant validity failure via the Fornell–Larcker criterion (Table 3). Disattenuation of the DM–DS and SM–SS correlations by reliability coefficients yields latent factor correlations at or above unity, and a competing two-factor model (deep vs. surface) fits the data equivalently [Δχ^2^(5) = 3.12, *p* = .681]. Researchers applying the ILAS to pre-professional samples should consider two-dimensional scoring (DM + DS composite; SM + SS composite) as a more defensible alternative. Future studies should examine: (a) whether this collapse replicates in conservatoire or university samples; (b) whether bifactor or higher-order SEM models provide additional explanatory value; and (c) whether supplementing self-report measures with behavioral indicators resolves the discriminant validity limitations (Bonneville-Roussy & Bouffard, 2015; Ericsson, 2006).

In summary, the present study makes three empirically defensible and theoretically meaningful contributions: (1) it demonstrates that the ILAS four-factor model collapses to a two-dimensional structure in pre-professional music students—a novel psychometric finding that advances domain-specific applications of SAL instruments; (2) it establishes that daily practice time is independently and negatively associated with surface strategy (β = −0.085, *p* = .012) after controlling for surface motive, providing evidence that practice habits carry a modest but distinct link to strategic orientation beyond motivational orientation alone; and (3) it documents small but statistically significant group differences by gender (d = 0.25–0.26) and grade level (η_p_^2^ = 0.03), interpreted as cohort effects warranting longitudinal investigation. Collectively, these findings have direct implications for ILAS scale validation, music pedagogy design, and future longitudinal and mixed-methods research in the domain of pre-professional music education.

### 5.2. Educational and Practical Implications

The present findings offer several implications for music pedagogy, particularly within the context of vocational music education, such as Fine Arts High Schools:

1. Prioritizing Motivation over Quantity: The strong association between motivational orientations and learning strategies suggests that teachers should prioritize fostering intrinsic curiosity and meaning-oriented engagement rather than solely pushing for increased practice hours (Hallam, 2002). Instructional methods that encourage students to reflect on the musical structure and personal interpretation may promote deeper strategic engagement (Nielsen, 2004).
2. Strategic Quality in Practice: The identified non-linear relationship between practice duration and strategy use implies that educators should move beyond the “more is better” narrative. Instead, creating structured and self-regulated practice environments that promote goal-setting, monitoring, and cognitive awareness may protect students from falling into rote-based, mechanical repetition during long practice sessions (Bonneville-Roussy & Bouffard, 2015; Zimmerman, 2002).
3. Fostering Autonomy: Since motivational factors emerged as the primary associates of strategy use, pedagogical approaches that support student autonomy and self-efficacy are likely to be more sustainable (Pintrich & Schunk, 2002). Rather than external pressure, developing a student’s internal “why” for learning appears to be the most effective way to enhance the “how” of their strategic practice.

## 6. Limitations and Suggestions for Future Research

### 6.1. Limitations of the Study

Several constraints must be acknowledged when interpreting the findings of this research:

- Scope and Sampling: This study is limited to students in Fine Arts High Schools in Türkiye. Due to the non-probability, volunteer-based sampling strategy and unequal subgroup sizes (e.g., 72.9% female), the findings may not be fully representative of the broader population of pre-professional musicians (Creswell & Creswell, 2018).
- Self-Report Bias: The data were derived from self-report scales, which may be susceptible to social desirability bias or idealized reporting of practice behaviors (Podsakoff et al., 2003).
- Cross-Sectional Design: The study utilized a cross-sectional design, which precludes any inference regarding causal direction or temporal ordering. Consequently, the observed differences across grade levels must be interpreted as cohort differences rather than developmental changes (Maxwell & Cole, 2007).
- Theoretical Boundaries: Although the study established conceptual links to self-regulation and cognitive processes, it did not include direct, objective measures of intelligence or cognitive functioning (McPherson, 1997). Therefore, interpretations regarding these constructs remain theoretical.

### 6.2. Suggestions for Future Research

Based on the findings and limitations, the following directions are proposed for future inquiry:

- Longitudinal Designs: Future studies should employ longitudinal tracking of student cohorts to clarify the developmental trajectories of learning approaches and to distinguish between cohort and aging effects (Fryer & Shum, 2022).
- Mixed-Method Approaches: Integrating quantitative findings with qualitative data, such as practice diaries, semi-structured interviews, or direct observations, could provide deeper insights into the “live” regulatory processes during instrumental practice (Johnson & Christensen, 2019). Specifically, qualitative categorizations and units of meaning could corroborate the quantitative associations identified in this study, offering a more nuanced understanding of how students conceptualize their strategic engagement.
- Quality and Quantity Research: Further investigation is needed to explore the thresholds of practice duration where cognitive fatigue begins to trigger surface strategies. Research should focus on the quality and structural organization of practice rather than its length.
- Advanced Modeling: Future research could utilize Structural Equation Modeling (SEM) to test more complex, indirect, or multivariate relationships among motivation, self-efficacy, and musical outcomes, thereby building a more comprehensive model of the musical learner (Hair et al., 2019).

## Figures and Tables

**Table 1:** Demographic characteristics of students.

Demographic Information		f	%
Gender	Female	242	72.89
	Male	90	27.11
	Total	332	100
Grade	9	72	21.69
	10	103	31.02
	11	70	21.08
	12	87	26.20
	Total	332	100
School	Mersin Nevit Kodallı FAHS	109	32.83
	İstanbul Aydın Doğan FAHS	68	20.48
	Trabzon Akçaabat FAHS	50	15.06
	Ankara FAHS	55	16.57
	Muğla FAHS	16	4.82
	Mardin FAHS	17	5.12
	Malatya Abdulkadir Eriş FAHS	17	5.12
	Total	332	100
Instrument Type	Bağlama	39	11.75
	Violin	96	28.92
	Piano	16	4.82
	Viola	27	8.13
	Cello	33	9.94
	Flute	39	11.75
	Kanun	11	3.31
	Guitar	43	12.95
	Other	28	8.43
	Total	332	100
Total duration of instrument playing experience	0–1 year	94	28.31
	1–2 years	81	24.40
	2–3 years	44	13.25
	3–4 years	45	13.55
	4+	68	20.48
	Total	332	100
Daily practice time	0–1 h	108	32.53
	1–2 h	122	36.75
	2–3 h	59	17.77
	3–4 h	23	6.93
	4+	20	6.02
	Total	332	100

**Table 4:** Confirmatory Factor Analysis (CFA) Fit Indices for the ILAS Four-Factor Model.

Index	Value	Threshold/Criteria	Interpretation
χ^2^	422.36	-	
df	224	-	
χ^2^/df	1.886	≤3.0 (Kline, 2016)	Acceptable
RMSEA	0.054	≤0.060 (Hu & Bentler, 1999)	Acceptable
90% CI for RMSEA	[0.046, 0.063]	-	
CFI	0.930	≥0.90 (Hu & Bentler, 1999)	Acceptable
TLI	0.921	≥0.90 (Hu & Bentler, 1999)	Acceptable
SRMR	0.059	≤0.080 (Kline, 2016)	Acceptable

Model: χ^2^(224) = 422.36, *p* < .001; χ^2^/df = 1.886; RMSEA = 0.054 [90% CI: 0.046, 0.063]; CFI = 0.930; TLI = 0.921; SRMR = 0.059.

**Table 5:** Descriptive statistics and reliability coefficients for the ILAS sub-dimensions.

Sub-Dimensions	Min.	Max.	Mean	SD	Skewness	Kurtosis	α
Deep Motive (DM)	1.00	5.00	3.57	1.19	−0.47	−0.15	0.79
Deep Strategy (DS)	1.00	5.00	3.47	1.21	−0.49	−0.09	0.87
Surface Motive (SM)	1.00	4.50	1.84	1.17	1.36	1.43	0.82
Surface Strategy (SS)	1.00	5.00	2.09	1.20	1.08	1.06	0.65

**Table 6:** Descriptive Statistics for ILAS Sub-dimensions.

Sub-Dimensions	N	M	SD	Level *
Deep Motive	332	3.57	1.19	Frequently
Deep Strategy	332	3.47	1.21	Frequently
Surface Motive	332	1.84	1.17	Rarely
Surface Strategy	332	2.09	1.20	Rarely

* Never: 1.00–1.80; Rarely: 1.81–2.60; Occasionally: 2.61–3.40; Frequently: 3.41–4.20; Always: 4.21–5.00.

**Table 7:** ANOVA results show differences in mean scores for ILAS sub-dimensions based on school attended.

Sub-Dimensions	FAHS	N	M	SD	F	*p*	η_p_^2^
Deep Motive	Mersin Nevit Kodallı	109	3.55	0.91	2.12	.050	0.04
	İstanbul Aydın Doğan	68	3.55	0.87			
	Trabzon Akçaabat	50	3.27	0.92			
	Ankara	55	3.74	0.84			
	Muğla	16	3.71	0.53			
	Mardin	17	3.58	0.82			
	Malatya Abdulkadir Eriş	17	3.99	0.68			
Deep Strategy	Mersin Nevit Kodallı	109	3.42	0.88	1.36	.230	0.02
	İstanbul Aydın Doğan	68	3.47	0.81			
	Trabzon Akçaabat	50	3.28	0.89			
	Ankara	55	3.66	0.83			
	Muğla	16	3.60	0.65			
	Mardin	17	3.34	0.68			
	Malatya Abdulkadir Eriş	17	3.73	0.77			
Surface Motive	Mersin Nevit Kodallı	109	1.85	0.86	0.93	.476	0.02
	İstanbul Aydın Doğan	68	1.92	0.93			
	Trabzon Akçaabat	50	1.86	0.86			
	Ankara	55	1.71	0.68			
	Muğla	16	1.82	0.55			
	Mardin	17	1.57	0.60			
	Malatya Abdulkadir Eriş	17	2.11	1.21			
Surface Strategy	Mersin Nevit Kodallı	109	2.09	0.91	1.29	.260	0.02
	İstanbul Aydın Doğan	68	2.18	0.99			
	Trabzon Akçaabat	50	2.16	0.81			
	Ankara	55	1.92	0.76			
	Muğla	16	2.04	0.67			
	Mardin	17	1.80	0.90			
	Malatya Abdulkadir Eriş	17	2.49	1.41			

Note. M = arithmetic mean (item mean score on a 1–5 scale); SD = standard deviation; η_p_^2^ = partial eta-squared. Significant differences are defined at *p* < .05.

**Table 8:** ANOVA Results for ILAS Sub-dimensions Based on Grade Level.

Sub-Dimensions	Grade	N	M	SD	F	*p*	η_p_^2^	Post Hoc
Deep Motive	9 ^a^	72	3.72	0.73	2.90	.035	0.03	a > c
	10 ^b^	103	3.66	0.85				
	11 ^c^	70	3.34	0.90				
	12 ^d^	87	3.53	0.95				
Deep Strategy	9	72	3.60	0.71	1.57	.196	0.01	-
	10	103	3.49	0.88				
	11	70	3.30	0.88				
	12	87	3.48	0.85				
Surface Motive	9	72	1.68	0.78	2.19	.089	0.02	-
	10	103	1.77	0.72				
	11	70	1.91	0.86				
	12	87	1.99	0.99				
Surface Strategy	9	72	1.99	0.89	1.13	.337	0.01	-
	10	103	2.02	0.80				
	11	70	2.15	1.01				
	12	87	2.22	0.98				

Note. M = arithmetic mean (item mean score on a 1–5 scale); SD = standard deviation; η_p_^2^ = partial eta-squared. Post hoc comparisons were conducted using the Scheffé test. Superscripts a–d correspond to the significant group differences (*p* < .05) detailed in the Post Hoc column.

**Table 9:** T-test Results for ILAS Sub-dimensions Based on Gender.

	Female (*n* = 242)		Male (*n* = 90)		t	*p*	d
Sub-Dimensions	M	SD	M	SD			
Deep Motive	3.52	0.91	3.73	0.76	−2.13	.035	0.25
Deep Strategy	3.41	0.84	3.63	0.49	−2.07	.039	0.26
Surface Motive	1.83	0.81	1.88	0.94	−0.56	.573	0.07
Surface Strategy	2.06	0.85	2.18	1.07	−1.07	.286	0.13

Note. M = arithmetic mean (item mean score on a 1–5 scale); SD = standard deviation; d = Cohen’s d effect size. Significant differences are accepted at *p* < .05.

**Table 10:** ANOVA Results for ILAS Sub-dimensions Based on Instrument Type.

Sub-Dimensions	Instrument Type	N	M	SD	F	*p*	η_p_^2^
Deep Motive	Bağlama	39	3.43	0.97	0.97	.461	0.02
	Violin	96	3.50	0.86			
	Piano	16	3.54	0.74			
	Viola	27	3.42	1.11			
	Cello	33	3.74	0.80			
	Flute	39	3.71	0.99			
	Kanun	11	4.02	0.64			
	Guitar	43	3.63	0.79			
	Other	28	3.53	0.69			
Deep Strategy	Bağlama	39	3.38	0.91	0.99	.442	0.02
	Violin	96	3.41	0.89			
	Piano	16	3.62	0.64			
	Viola	27	3.37	0.91			
	Cello	33	3.60	0.76			
	Flute	39	3.55	0.88			
	Kanun	11	3.98	0.97			
	Guitar	43	3.48	0.77			
	Other	28	3.34	0.80			
Surface Motive	Bağlama	39	1.95	0.88	1.33	.229	0.03
	Violin	96	1.93	0.88			
	Piano	16	1.59	0.75			
	Viola	27	2.00	0.92			
	Cello	33	1.77	0.90			
	Flute	39	1.60	0.65			
	Kanun	11	2.06	1.26			
	Guitar	43	1.67	0.63			
	Other	28	1.97	0.87			
Surface Strategy	Bağlama	39	2.22	0.99	1.23	.283	0.03
	Violin	96	2.11	0.94			
	Piano	16	1.73	0.89			
	Viola	27	2.09	0.88			
	Cello	33	2.14	1.04			
	Flute	39	1.79	0.57			
	Kanun	11	2.33	1.32			
	Guitar	43	2.11	0.78			
	Other	28	2.30	1.02			

Note. M = arithmetic mean (item mean score on a 1–5 scale); SD = standard deviation; η_p_^2^ = partial eta-squared. Significant differences are defined at *p* < .05.

**Table 11:** ANOVA Results for ILAS Sub-dimensions Based on Duration of Playing the Instrument.

Sub-Dimensions	Duration (Years)	N	M	SD	F	*p*	η_p_^2^
Deep Motive	0–1	94	3.51	0.86	1.00	.410	0.01
	1–2	81	3.53	0.89			
	2–3	44	3.51	0.88			
	3–4	45	3.80	0.89			
	4+	68	3.61	0.86			
Deep Strategy	0–1	94	3.43	0.83	2.06	.086	0.02
	1–2	81	3.33	0.83			
	2–3	44	3.37	0.87			
	3–4	45	3.65	0.84			
	4+	68	3.64	0.83			
Surface Motive	0–1	94	1.80	0.76	0.69	.600	0.01
	1–2	81	1.89	0.85			
	2–3	44	1.85	0.84			
	3–4	45	1.99	1.11			
	4+	68	1.74	0.76			
Surface Strategy	0–1	94	2.05	0.86	1.09	.360	0.01
	1–2	81	2.15	0.79			
	2–3	44	2.20	0.94			
	3–4	45	2.23	1.13			
	4+	68	1.93	0.95			

Note. M = arithmetic mean (item mean score on a 1–5 scale); SD = standard deviation; η_p_^2^ = partial eta-squared. Significant differences are defined at *p* < .05.

**Table 12:** ANOVA Results for ILAS Sub-dimensions Based on Daily Practice Time.

Sub-Dimensions	Practice Time (h)	N	M	SD	F	*p*	η_p_^2^	Post Hoc
Deep Motive	0–1 ^a^	94	3.13	0.88	15.58	<.001	0.16	a < b, c, d, e
	1–2 ^b^	81	3.68	0.83				b < d
	2–3 ^c^	44	3.71	0.74				
	3–4 ^d^	45	4.24	0.54				
	4+ ^e^	68	4.16	0.61				
Deep Strategy	0–1 ^a^	94	3.01	0.88	20.34	<.001	0.20	a < b, c, d, e
	1–2 ^b^	81	3.61	0.73				b, c < d, e
	2–3 ^c^	44	3.53	0.70				
	3–4 ^d^	45	4.16	0.56				
	4+ ^e^	68	4.17	0.60				
Surface Motive	0–1 ^a^	94	2.07	0.87	3.80	0.037	0.027	a > b, c
	1–2 ^b^	81	1.71	0.80				
	2–3 ^c^	44	1.68	0.63				
	3–4 ^d^	45	1.68	0.97				
	4+ ^e^	68	2.02	1.20				
Surface Strategy	0–1 ^a^	94	2.36	0.89	5.18	<.001	0.06	a > b, c, d
	1–2 ^b^	81	1.97	0.85				
	2–3 ^c^	44	1.88	0.74				
	3–4 ^d^	45	1.77	1.02				
	4+ ^e^	68	2.38	1.33				

Note. M = arithmetic mean (item mean score on a 1–5 scale); SD = standard deviation; η_p_^2^ = partial eta-squared. Significant differences are defined at *p* < .05. Post hoc comparisons were conducted using the Scheffé test. Superscripts a–e correspond to the significant group differences (*p* < .05) detailed in the Post Hoc column.

**Table 13:** Pearson Correlation Coefficients Among ILAS Sub-dimensions and DPT.

Variables	DM	DS	SM	SS	DPT
Deep Motive (DM)	1				
Deep Strategy (DS)	0.836	1			
Surface Motive (SM)	−0.223	−0.161 *	1		
Surface Strategy (SS)	−0.202	−0.115	0.789	1	
Daily Practice Time (DPT)	0.409	0.386	−0.081	−0.149	1

Note. *p* < .001, * *p* < .05. All values are based on item mean scores (1–5 scale). Significant correlations are indicated in bold. Diagonal values represent the correlation of each variable with itself.

**Table 14:** Regression-Based Associations with DS (Strategic Depth).

Model	Predictor Variable	B	SE	β	t	*p*	R^2^	F
Model 1	Constant	0.584	0.137		4.25	<.001	0.702	387.59
	Deep Motive	0.775	0.031	0.815	24.70	<.001		
	Daily Practice Time	0.040	0.024	0.054	1.63	.104		

Note. B = unstandardized coefficient; SE = standard error; β = standardized coefficient; R^2^ = coefficient of determination; Adj. R^2^ = 0.700; DW = 2.039. *p* < .05. *p* < .001. The contribution of DPT was not statistically significant (*p* = .104) after controlling for DM, indicating that practice duration does not account for independent variance in DS once motivational orientation is controlled.

**Table 15:** Regression-Based Associations with Surface Strategy (SS).

Model	Predictor Variable	B	SE	β	t	*p*	R^2^	F
Model 2	Constant	0.673	0.099		6.81	<.001	0.631	280.75
	Surface Motive	0.855	0.037	0.783	23.28	<.001		
	Daily Practice Time	−0.070	0.027	−0.085	−2.54	.012		

Note. B = unstandardized coefficient; SE = standard error; β = standardized coefficient; R^2^ = coefficient of determination; Adj. R^2^ = 0.628; DW = 1.996. *p* < .05. *p* < .001. All associations are correlational; no causal inference is implied. VIF values ≤ 1.01 across all predictors; multicollinearity was not a concern in this model.

## Data Availability

The data supporting the findings of this study are included within the article.

## Abbreviations

The following abbreviations are used in this manuscript:

ILAS	Instrument Learning Approaches Scale
FAHS	Fine Arts High School
DM	Deep Motive
DS	Deep Strategy
DL	Deep Learning
SS	Surface Strategy
SM	Surface Motive
SL	Surface Learning
SAL	Student Approaches to Learning
SLA	Surface Learning Approach
DLA	Deep Learning Approach
